# Internet addiction influences life satisfaction through social support among Chinese college students: a moderated mediation model of grit

**DOI:** 10.3389/fpsyg.2025.1654839

**Published:** 2025-10-22

**Authors:** Tingting Ma, Jianan Li, Chang Seek Lee

**Affiliations:** ^1^School of Digital Economics and Management, Software Engineering Institute of Guangzhou, Guangzhou, China; ^2^Department of Lifelong Education, Hanseo University, Seosan, Republic of Korea

**Keywords:** internet addiction, social support, life satisfaction, grit, Chinese college students

## Abstract

The prevalence of Internet addiction among college students has gained significant attention in recent years. Research has established a negative relationship between Internet addiction and life satisfaction, although the underlying mechanisms are not fully understood. The present study aims to examine the relationship between Internet addiction, grit, social support, and life satisfaction. A random sampling method was used to recruit 304 Chinese college students to complete a questionnaire that included measures of Young’s Internet Addiction Scale, Multidimensional Scale of Perceived Social Support, Satisfaction with Life Scale, and 12-Item Grit Scale. For data analysis, SPSS, PROCESS macro and AMOS 23 were used to conduct confirmatory factor analysis, descriptive statistics analysis, reliability analysis, correlation analysis, and moderated mediation analysis. The results revealed that Internet addiction was negatively correlated with life satisfaction, and social support plays a mediating role between them. Moreover, grit moderated the mediation effect of social support in the relationship between Internet addiction and life satisfaction. This suggests that Internet addicts with higher levels of grit are less likely to experience a significant decline in social support. The study provides a deeper insight into the mechanisms through which Internet addiction hampers life satisfaction, suggesting that the influence may be channeled through social support. However, fostering a strong sense of grit could serve as a protective factor against the adverse impacts of internet addiction on life satisfaction. The broader implications for both research and practical applications in the field are subsequently elaborated.

## 1 Introduction

According to the 54th Statistical Report on Internet Development in China by China Internet Network Information Center, college students have become one of the main Internet user groups (CNNIC, 2024). With the popularization of mobile Internet, college students’ Internet use has penetrated into their daily lives, from academic learning (e.g., online courses, literature searches) to social interactions (e.g., instant messaging, social media) and entertainment (e.g., video streaming, online games). While moderate Internet use brings convenience, excessive and uncontrollable use has gradually evolved into a prominent issue. Prior national surveys of Chinese college students show that the detection rate of Internet addiction ranges from 11 to 13.6% (Shao et al., 2018; Hsieh et al., 2019). As a condition defined by an individual’s inability to control their Internet use, Internet addiction has been repeatedly reported to be linked to negative outcomes over the years (Young, 1998; Shek et al., 2015). Specifically, scholars have found that Internet addiction can eventually lead to a variety of problems, including psychological, social, academic/occupational problems, and functioning impairment (Young, 2004; Azwa Ambad et al., 2017; Baturay and Toker, 2019).

To understand the adverse impacts of Internet addiction, a large body of existing research has frequently incorporated life satisfaction as a key variable to examine its relationship with Internet addiction. Life satisfaction, as the cognitive component of subjective well-being (SWB), was considered a reliable and stable indicator that reflects one’s overall health and well-being, and research on college students specifically highlights how this indicator links to key areas of their daily functioning (Diener, 2000; Raphael, 1996). Many studies claimed that higher life satisfaction of college students was correlated with positive academic outcomes (Antaramian, 2017), while lower life satisfaction was the cause of a variety of psychological and social problems (Sun and Shek, 2010). Unsurprisingly, enhancing students’ life satisfaction has long been recognized as a critical educational goal (O'Neill, 1981). However, consistent findings highlight that Internet addiction exerts a significant negative effect on life satisfaction. For example, a cross-national meta-analysis showed that Internet addiction is inversely associated with quality of life, and life satisfaction serves as a key subjective reflection of this association (Cheng and Li, 2014). Since college students are particularly vulnerable to Internet addiction (Kandell, 1998; Young, 2004), it is urgent to investigate the underlying mechanisms through which Internet addiction undermines life satisfaction, as well as identifying potential protective factors that can mitigate this impact.

Perceived social support refers to an individual’s perceptions of general support or specific supportive behaviors (available or enacted upon) from people in their social network (Malecki and Demaray, 2002). Numerous findings have confirmed that perceived social support from family and faculty is an important predictor of life satisfaction among college students, because during the college years, students may need various kinds of support when facing challenging life events (Yalçın, 2011). However, excessive Internet use could lead to reduced and disrupted social relationships in reality, and therefore decrease individuals’ perceived social support (Young and Rogers, 1998; Swickert et al., 2002). Based on the previous research, we hypothesized that social support mediates the relationship between Internet addiction and life satisfaction.

Furthermore, despite the reported adverse effects of Internet addiction on college students, these effects may vary by individual differences (Menon et al., 2018; Khosravi et al., 2022). Researchers found that college students with higher grit were more likely to confront difficulties and challenges, making them more sensible to the support available from their social networks. Moreover, grit helps students foster greater trust in others, thereby improving their comprehension of social support (Du et al., 2024). Other studies also revealed a positive correlation between grit and social support (Yang and Wu, 2021; Ma et al., 2024). In this case, it is speculated that grit moderates the mediating effect of social support between Internet addiction and life satisfaction. Nevertheless, currently there is no empirical investigation of such results. Therefore, this study aims to examine the mediating role of social support between Internet addiction and life satisfaction, and test the moderating role of grit. This study enriches the existing literature by exploring how a personal trait offsets the negative impact of Internet addiction in a psychological mechanism. The research questions are set as follows. First, what are the correlations between Internet addiction, grit, social support, and life satisfaction? Second, does grit moderate the mediation effect of social support between Internet addiction and life satisfaction?

## 2 Theoretical background

### 2.1 Internet addiction and life satisfaction

Internet addiction refers to a psychological dependence on the Internet, regardless of the type of activity once logged on (Kandell, 1998). Symptoms often include a large investment of time, energy, and money on Internet activities, inability to control Internet usage, experiencing unpleasant feelings (e.g., anxiety, depression, emptiness, and loneliness) when offline, and isolation from families and friends in reality (Davis, 2001). Moreover, those deemed Internet addicts reported more negative consequences on their daily lives than non-addicts, including relationship breakdowns, academic or occupational problems, and health problems in many studies (Young, 1998; Chou and Hsiao, 2000). In general, Internet addiction is considered a behavioral disorder that can create undesirable outcomes.

Life satisfaction was defined as the degree to which a person positively evaluates the overall quality of his/her life as-a-whole (Veenhoven, 1996). It is often studied as the best indicator of an individual’s perceived life quality (Huebner et al., 2006), with influential factors including finding life meaningful, developing a positive individual identity, maintaining physical well-being, achieving economic security, and nurturing social relationships, all of which align with the “valuable resources” highlighted in Conservation of Resources (COR) Theory (Zisselman and Cutillo-Schmitter, 1999; Hobfoll, 1989). COR Theory posits that individuals actively strive to acquire, retain, and protect such resources (e.g., social connections, physical health, academic/occupational success), and Internet addiction can be understood through this lens as a behavior that depletes these key assets: excessive online engagement diverts time and energy from fostering real-world relationships, which in turn leads to social isolation; it also hinders academic or occupational performance because of neglected responsibilities and undermines physical health through disrupted sleep or sedentary habits. As life satisfaction depends on the availability of these resources, resource depletion from Internet addiction directly reduces life satisfaction.

Also, the causal relationship between Internet addiction and life satisfaction has been extensively investigated across different populations, particularly young people, and empirical findings consistently confirm a significant negative causal link between the two (Cao et al., 2011; Shahnaz and Karim, 2014; Şahin, 2016). Further supporting this link, researchers studied 20 college students with problematic internet use (screened from 418 undergraduates) using a reality therapy-based group counseling program. They found that the program not only curbed addictive behaviors but also boosted life satisfaction (Odacı and Çelik, 2017). This finding indirectly reflects how mitigating resource depletion, via reduced Internet addiction, can restore conditions for greater life satisfaction. However, research on the underlying mechanism through which Internet addiction affects life satisfaction, including how specific resource losses (e.g., social disconnection) mediate this relationship, remains insufficient and inconclusive.

### 2.2 Social support as a mediator

Perceived social support involves an evaluation or appraisal of whether and to what extent an interaction, pattern of interactions, or relationship is helpful (Schaefer et al., 1981). For young people, social support from family, peers, and teachers is regarded as especially beneficial, which enhances their functioning and/or may buffer them from adverse outcomes (Demaray and Malecki, 2002; Malecki and Demaray, 2003).

Several studies have shown that perceived social support is associated with Internet addiction and life satisfaction, respectively (Naseri et al., 2015; Quinones and Kakabadse, 2015; Shahyad et al., 2011). In traditional social exclusion theory, Internet addicts are at a higher risk of losing their social ties with their family, friends, and significant others offline (Bagir et al., 2020). When individuals form online relationships, the time they spend with real-life people will eclipse, and over time quality of once stable relationships will be hurt and broken (Young, 1999). As a result, the inadequacy of supportive ties would decrease perception of social support (Barrera Jr, 1986).

In empirical studies, such a negative correlation between Internet addiction and perceived social support has been found repeatedly (Çevik and Yildiz, 2017; Tan, 2019). Furthermore, perceived social support has been verified as the strongest predictor of life satisfaction across the literature, which means lower perceived social support will eventuate in a lower level of life satisfaction (Cohen and Wills, 1985; Kasprzak, 2010).

Furthermore, from a COR Theory perspective, social support is a critical “social resource” that buffers against stress and enhances life satisfaction (Cohen and Wills, 1985). Internet addiction reduces this resource (by displacing offline relationships), and lower social support, in turn, reduces life satisfaction (as individuals lack resources to cope with college stressors). This forms the mediating path: Internet addiction leads to reduced social support (which can be viewed as resource loss), and reduced social support further results in lower life satisfaction.

Based on the previous results, it can be inferred that social support mediates between Internet addiction and life satisfaction. Although the mediating effect of social support has been verified in other similar studies (Guo et al., 2021; Wang and Fu, 2024), none of them explored the relationship between Internet addiction and life satisfaction through social support mediation. This study intends to fill this gap.

### 2.3 Grit as a moderator

Grit, defined as perseverance and passion for long-term goals, is an important positive psychological trait that reflects an individual’s self-motivation, self-discipline, and self-adjustment (Duckworth et al., 2007). Gritty people are able to seek practical alternatives when their goals or actions appear unattainable within their realm of passionate interest (Duckworth and Gross, 2014). Meanwhile, they tend to garner attention and support available to them in the process of finding feasible solutions and thus have a higher level of perceived social support (Du et al., 2024). Moreover, several studies supported that there was a positive association between grit and perceived social support among adolescents, high school students, college students, and nurses (Kim and Lee, 2022; Clark et al., 2019; Du et al., 2024; Liu et al., 2024). Notably, this capacity for maintaining social support may be particularly relevant in the context of Internet addiction. Grittier people are more likely to maintain social connections despite internet addiction (e.g., scheduling time for friends instead of uncontrolled online use), thus weakening the mediating effect of social support that would otherwise link Internet addiction to reduced life satisfaction. While prior research has substantiated basic relationships between these variables, it overlooks how grit moderates the Internet addiction-social support-life satisfaction pathway among college students. Therefore, this study addresses this gap by testing grit’s moderated mediation role.

## 3 Research methods

### 3.1 Research model

**Figure 1:**
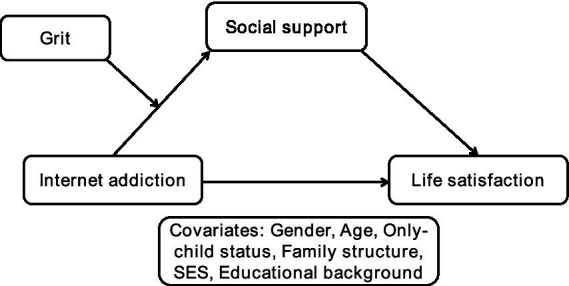
Conceptual model.

The conceptual model of the moderated mediation effect of grit on social support between Internet addiction and life satisfaction was established in Figure 1, using Model No.7 developed by Hayes (2013) in SPSS PROCESS macro ver.4.2.

### 3.2 Participants and procedures

A total of 304 college students from 32 provinces of China participated in this study. In the survey, 40.1% of the participants were male and 59.9% were female, ranging from age 18 to 24 (SD = 1.144). 58.6% of the respondents were only children, while 41.4% had siblings. Regarding family structure, 8.2% were from single-parent families and 91.8% from two-parent families. Socioeconomically, 25.7% came from wealthy families, 53.6% from average families, and 20.7% from less wealthy families. In terms of educational background, 49.7% were 4-year undergraduate students and 50.3% were 3-year college students.

The data collection was done using simple random sampling from 870,000 Chinese college students on the Wenjuanxing platform, a large survey company in China. Initially, we defined the target population as college students across multiple universities to ensure representativeness. The Wenjuanxing platform then implemented its built-in random sampling algorithm to select participants from its large user pool of verified college students. After confirming the sample of 3,782 students through this sampling, the platform directly sent the questionnaire link to the selected students. Upon receiving the link, students could click on it to access the online survey. Prior to questionnaire completion, they were required to read and confirm an informed consent form, which detailed the study purpose, confidentiality guarantee, voluntary participation right, and withdrawal terms. Only students who agreed to the consent could proceed to independently complete the questionnaire. The platform also imposed technical restrictions (e.g., one response per IP address) to avoid duplicate submissions from the same individual. In total, 1,323 students responded to the online questionnaires. After collecting completed responses, invalid questionnaires were defined and removed based on the following criteria: incomplete responses (i.e., unanswerable items left blank), careless responses with obvious logical inconsistencies (contradictory answers to related questions, or responses that violated basic logical coherence), failure to pass the attention-check questions randomly inserted by the Wenjuanxing system, and abnormally short or long response times (reasonable response time is about 3–5 s per question). Through this screening, 737 invalid responses were excluded, and the remaining 304 valid responses were ultimately used for statistical analysis. To encourage thoughtful and complete participation, participants who submitted fully valid questionnaires received a 5-yuan reward as compensation.

### 3.3 Measurement

#### 3.3.1 Internet addiction

Internet addiction was measured by the Chinese Internet Addiction Scale’s 10-item questionnaire (Young, 1998; Shek et al., 2008). Participants indicated whether they had experienced each form of Internet addiction in the past year by responding with “yes”(1 point) or “no”(0 point) to the corresponding items. The total score range is 0 to 10. An individual was identified as having “Internet addiction” if they reported experiencing four or more of the specified behaviors (scores ≥ 4). An example of the items is “Do you stay online longer than originally intended?.” The test has been widely applied in other Chinese studies and demonstrated strong reliability and validity. In this study, the Cronbach’s *α* for Internet addiction was 0.668. By convention, an alpha of 0.60 to 0.70 is often considered acceptable (Vaske, 2008).

#### 3.3.2 Social support

Social support was measured using the Chinese version of the Multidimensional Scale of Perceived Social Support (MSPSS), which has been widely tested with Chinese college students and has shown good reliability and validity (Jiang, 2001). The Confirmatory Factor Analysis conducted for the scale construct validity indicated the fit indices as follows: CFI = 1.00, GFI = 1.00, AGFI = 0.969, RMSEA = 0.00. The Cronbach’s α in this study for the MSPSS was 0.918. The scale includes three dimensions, each with four items. These dimensions assess perceived support from significant others, family, and friends. Respondents indicated their level of agreement on a 7-point Likert scale, ranging from 1 (very strongly disagree) to 7 (very strongly agree), with total scores potentially ranging from 12 to 84.

#### 3.3.3 Life satisfaction

The Chinese version translated by the authors, adapted from the Satisfaction with Life Scale (SWLS) (Diener et al., 1985), was used to assess college students’ life satisfaction. This scale comprises five statements, such as “I am satisfied with my life.” Participants were prompted to rate their satisfaction on a 7-point Likert scale, where 1 signifies strong disagreement and 7 indicates strong agreement. The total score range is 5 to 35, with higher scores indicating higher life satisfaction. This scale is widely applied, and the Chinese translation version has reached good reliability in the authors’ prior research (Ma and Lee, 2023). For this research, the scale’s Cronbach’s alpha reliability coefficient was 0.885. Confirmatory factor analysis for SWLS yielded good fit values: CFI = 0.998, GFI = 0.993, AGFI = 0.973, RMSEA = 0.035.

#### 3.3.4 Grit

The Chinese version of the 12-Item grit scale, translated by Xie et al. (2017), was used to measure the grit levels of participants. This scale was verified to have good validity in previous research (GFI = 0.957, NFI = 0.927, IFI = 0.965, RMSEA = 0.049) (Xie et al., 2017). Developed by Duckworth et al. (2007), this scale features 12 items divided into two subscales: Consistency of Interests (CI) and Perseverance of Effort (PE). Respondents evaluated the statements on a 5-point Likert scale, where 1 indicated “Not like me at all” and 5 signified “Very much like me.” Following the reversal of scores for the four items pertaining to Consistency of Interests (CI), an average score was determined to represent overall grit levels, with higher scores indicating greater grit. The total score range is 12 to 60. The Cronbach’s *α* reliability coefficient for this scale in our study was 0.853.

#### 3.3.5 Controlled variables

As for the controlled variables, we include gender (male/female), age, only-child status (yes/no), family structure (single parent/two parents), perceived socioeconomic status (very affluent/affluent/average/poor/very poor), and educational background (associate degree or below/bachelor’s degree/Master’s degree/doctorate) during the analyzing process.

### 3.4 Data analysis

We used SPSS PC + Win. Ver. 25.0, SPSS PROCESS macro Version 4.2, and AMOS 23 to analyze the data.

First, Given that all variables in this study were measured via self-reported questionnaires, potential common method bias (CMB) might arise from factors like response inertia, social desirability, and uniform measurement scenarios. To assess potential common method bias (CMB) we followed the approach recommended by Podsakoff et al. (2003) using CFA-based single-factor test in AMOS 23. If CMB is severe, all items will load predominantly on a single “common method factor” (CMV), and the fit of the single-factor model will be comparable to the “core construct model.” If the addition of CMV leads to a substantial improvement in fit indices (ΔCFI > 0.01, ΔRMSEA > 0.015), CMB is considered significant. During confirmatory factor analysis (CFA), all items loaded onto their theoretical constructs (IA, SS, LS, Grit) and a latent factor for common method variance (CMV). We compared fit indices of the “core construct model” (no CMV) and “model with CMV” to evaluate CMB impact.

Next, we calculated descriptive statistics for main variables and ran Spearman’s rank-order correlation analysis.

Then, we tested the mediating effect of social support via SPSS PROCESS Macro Model 4, and the moderated mediation effect of grit via Model 7. The bootstrap method (95% confidence level, 5,000 samples) verified mediation, and independent/moderating variables were mean-centered for moderated mediation.

## 4 Results

### 4.1 Common method biased analysis

The fitting results of the core construct CFA model (without CMV) and the model with CMV were presented in Table 1.

**Table 1:** Fit indices of the core construct CFA model.

Model	*χ* ^2^	df	*χ*^2^/df	GFI	CFI	RMSEA
Core construct model (IA, SS, LS, Grit)	1389.000	688	2.019	0.806	0.864	0.058
Model with CMV	1189.490	652	1.824	0.834	0.896	0.052

IA, internet addiction; SS, social support; LS, life satisfaction.

As shown in Table 1, the fit of the model with CMV was only slightly better than the core construct model, with ΔCFI = 0.032 and ΔRMSEA = 0.006 (one below the threshold for significant CMB). Although ΔCFI (0.032) slightly exceeded the conventional threshold of 0.01, the absolute fit indices of both models remained reasonably acceptable, and the incremental improvement after adding CMV was modest. Thus, CMB was unlikely to substantially bias the results.

### 4.2 Descriptive analysis and correlations between variables

The results of the descriptive analysis and Spearman’s rank-order correlation analysis were presented in Table 2, 3. According to the results of descriptive statistics, 52.6% out of 304 college students were identified as addicted to the Internet, with a cutoff point at 4. The mean score of Internet addiction was 3.62 (SD = 2.29). For social support, life satisfaction, and grit, the mean values were 5.22 (SD = 1), 4.28 (SD = 1.33), and 3.29 (SD = 0.65), respectively.

**Table 2:** Frequency of Internet addiction.

Internet addiction	*N*	%
Addictive	160	52.6
Not addictive	144	47.4
Total	304	100

**Table 3:** Correlation and descriptive statistics analysis results.

Variables	*M*	SD	1	2	3	4
1. Internet addiction	3.62	2.29	1			
2. Social support	5.22	1.00	−0.34^***^	1		
3. Life satisfaction	4.28	1.33	−0.39^***^	0.66^***^	1	
4. Grit	3.29	0.65	−0.38^***^	0.60^***^	0.53^***^	1

*N* = 304. ^***^p < 0.001.

The results of the correlation analysis showed that Internet addiction had a negative correlation with social support (*r* = −0.34, *p* < 0.001), life satisfaction (*r* = −0.39, *p* < 0.001), and grit (*r* = −0.38, *p* < 0.001). On the other hand, positive correlations between social support and life satisfaction (*r* = 0.66, *p* < 0.001), social support and grit (*r* = 0.60, *p* < 0.001), and life satisfaction and grit (*r* = 0.53, *p* < 0.001) were found. In addition, since the correlation coefficients were all lower than 0.7, no multicollinearity problem was found.

### 4.3 Mediation effect of social support

As indicated by the results of mediation effect analysis in Table 4, Internet addiction was negatively associated with social support (*B* = −0.089, *p* < 0.001). In the dependent variable model, the relationship between Internet addiction and life satisfaction was significant and negative (*B* = −0.075, *p* < 0.01), while social support contributed to life satisfaction significantly positively (*B* = 0.619, *p* < 0.001). In addition, the indirect effect of social support was −0.055 (95% CI = [−0.089, −0.026]), showing no 0 between the upper and lower limits of bootstrap. Therefore, the mediating role of social support between Internet addiction and life satisfaction was confirmed.

**Table 4:** Mediation analysis.

Variables		Mediating variable model (DV: social support)			Dependent variable model (DV: life satisfaction)		
		*B*	SE	*t*	*B*	SE	*t*
Constant		6.804	0.503	13.533^***^	1.974	0.679	2.906^**^
IV	Internet addiction	−0.089	0.022	−4.016^***^	−0.075	0.024	−3.072^**^
MV	Social support	–	–	–	0.619	0.062	10.027^***^
CV	Gender	0.131	0.103	1.276	−0.021	0.110	−0.193
	Age	0.078	0.044	1.765	0.077	0.047	1.626
	Only-child status	−0.108	0.105	−1.036	−0.132	0.111	−1.186
	Family structure	0.034	0.184	0.183	0.466	0.195	2.390^*^
	SES	−0.533	0.067	−7.912^***^	−0.458	0.079	−5.817^***^
	Educational background	0.009	0.101	0.091	−0.099	0.107	−0.927
Model summary	*R* ^2^	0.291			0.554		
	*F*	17.337^***^			45.854^***^		

	Effect	SE	95% Confidence interval	
			LLCI	ULCI
Total effect	−0.130	0.027	−0.184	−0.076
Direct effect	−0.075	0.024	−0.122	−0.027
Indirect effect	−0.055	0.016	−0.089	−0.026

IV, independent variable; MV, mediating variable; DV, dependent variable; CV, covariates; B, unstandardized regression coefficient; t, *t*-value; ^*^p < 0.05, ^*^p < 0.01, ^***^p < 0.001; *N* = 304, bootstrap sample size = 5,000; LLCI, lower bootstrap value; ULCI, upper Bootstrap value.

### 4.4 Moderated mediation effect of grit

The results of the moderated mediation effect analysis were demonstrated in Table 5.

**Table 5:** Moderated mediation analysis.

Variables		Mediating variable model (DV: social support)			Dependent variable model (DV: life satisfaction)		
		*B*	SE	*t*	*B*	SE	*t*
Constant		6.063	0.433	14.011^***^	1.704	0.662	2.575^*^
IV Internet addiction		−0.041	0.021	−1.986^*^	−0.075	0.024	−3.072^**^
M Grit		0.723	0.075	9.688^***^	–	–	–
IT Grit x Social support		0.086	0.029	3.003^**^	–	–	–
△*R*^2^		0.016^**^	–	–	–	–	–
MV Social support		–	–	–	0.619	0.062	10.027^***^
CV Gender		0.124	0.090	1.387	−0.021	0.110	−0.193
Age		0.018	0.039	0.455	0.077	0.047	1.626
Only-child status		−0.036	0.092	−0.387	−0.132	0.111	−1.186
Family structure		0.005	0.160	0.031	0.466	0.195	2.390^*^
SES		−0.358	0.062	−5.813^***^	−0.458	0.079	−5.817^***^
Educational background		0.051	0.088	0.577	−0.099	0.107	−0.927
Model summary	*R* ^2^	0.470			0.554		
	*F*	28.997^***^			45.854^***^		

Conditional effects of Internet addiction at values of grit					
Grit	Effect(B)	SE	*t*	LLCI	ULCI
−0.6484 (M − SD)	−0.096	0.030	−3.236^**^	−0.155	−0.038
0.0000 (M)	−0.041	0.021	−1.986^*^	−0.081	−0.000
0.6484 (M + SD)	0.015	0.025	0.583	−0.035	0.065

Conditional indirect effects (Internet addiction → social support → life satisfaction)				
Grit	Effect(B)	BootSE	BootLLCI	BootULCI
−0.6484 (M − SD)	−0.056	0.024	−0.105	−0.009
0.0000 (M)	−0.025	0.015	−0.053	0.006
0.6484 (M + SD)	0.009	0.012	−0.016	0.033

Index of moderated mediation				
	Index	BootSE	BootLLCI	BootULCI
Grit	0.053	0.019	0.015	0.088

IV, independent variable; MV, mediating variable; DV, dependent variable; IT, interaction term; CV, covariates; B, unstandardized regression coefficient; t, *t*-value; ^*^p < 0.05, ^*^p < 0.01, ^***^p < 0.001; *N* = 304, bootstrap sample size = 5,000; LLCI, lower bootstrap value within 95% confidence interval; ULCI, Upper Bootstrap value within 95% confidence interval.

**Figure 2:**
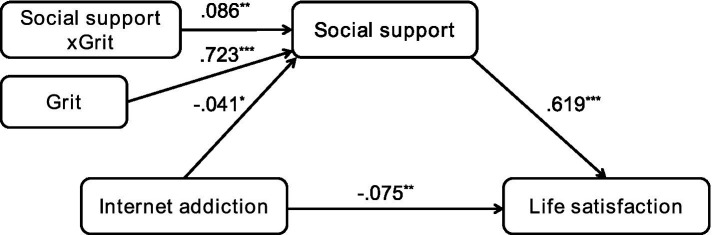
Moderating effect of grit in the statistical model. *p < 0.05, **p < 0.01, ***p < 0.001. All coefficients were unstandardized regression coefficients.

**Figure 3:**
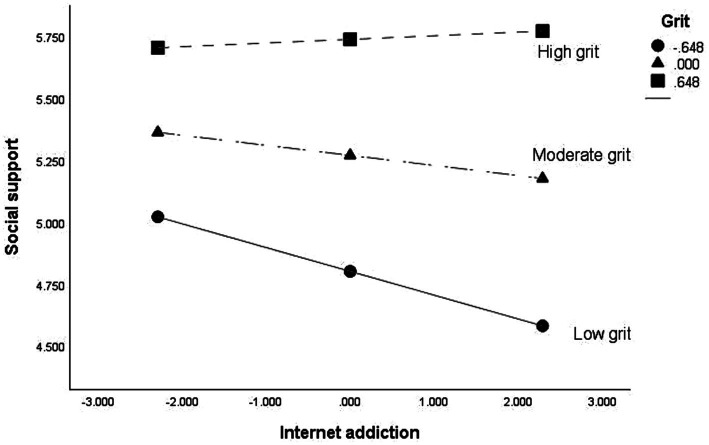
The moderating effect of grit in the graph. The numbers in the right corner caption (−0.648, 0.000, 0.648) represent the specific values of grit at which the conditional effect of internet addiction on social support is calculated. These values correspond to the percentile-based cut-offs for low, moderate, and high grit, respectively.

Internet addiction was negatively associated with social support (*B* = −0.041, *p* < 0.05) and life satisfaction (*B* = −0.075, *p* < 0.01), and social support was positively associated with life satisfaction (*B* = 0.619, *p* < 0.001). The interaction term between social support and grit was statistically significant (*B* = 0.086, *p* < 0.01), verifying the moderating effect of grit on the relationship between Internet addiction and social support (see Figure 2). To understand thoroughly, the moderating effects of grit at different levels were graphed in Figure 3. Internet addiction negatively influenced social support, particularly at lower grit levels (M − SD: *B* = −0.096, *p* < 0.01; M: *B* = −0.041, *p* < 0.05), as shown by a significant decline in social support with increased Internet addiction. However, this effect diminished and became non-significant at higher grit levels (M + SD: *B* = 0.015, *p* > 0.05), indicating that individuals with greater grit might be more resilient to the negative social impacts of Internet addiction.

Furthermore, according to the results of conditional indirect effect analysis, the conditional indirect effects of Internet addiction on life satisfaction through social support were various at three grit levels. At the M − SD grit level, a significant negative moderated mediation effect was observed, marked by an effect size of *B* = −0.056 with a 95% CI of [−0.105, −0.009], excluding zero and highlighting the substantial impact of Internet addiction on life satisfaction via social support. In contrast, at the mean (M) grit level, the indirect effect, though negative at *B* = −0.025, failed to reach statistical significance as the 95% CI of [−0.053, 0.006] includes zero. This pattern continued at the M + SD grit level, where the indirect effect, despite being positive at *B* = 0.009, did not achieve statistical significance with a 95% CI of [−0.016, 0.033] that also included zero. Collectively, these findings indicated that the indirect effect of Internet addiction on life satisfaction through social support was significant only at the lower end of the grit spectrum. Lastly, a moderated mediation index of 0.0530 without 0 between the 95% CI indicated the significance of the moderated mediation effect of grit. Given these results, the moderated mediating effect of grit was verified in the path from Internet addiction to life satisfaction through social support.

## 5 Discussion

The present study investigated the relationship between Internet addiction, grit, social support, and life satisfaction. Findings indicated that social support mediated the influence of Internet addiction on life satisfaction. In addition, grit moderated the mediation effect of social support in the relationship between Internet addiction and life satisfaction.

Firstly, the prevalence of Internet addiction was detected as 52.6%, which accounted for more than half of the respondents. This result was higher than the reported rate of 41.84% in a meta-analysis research among Asian college students (Liu et al., 2025). Although the difference may be due to the sample size, we should not ignore the fact that the prevalence rate of Internet addiction has been growing significantly during recent years. From 2014 to 2021, the reported detection rate ranged from 12.8 to 24.3% (Zhou et al., 2014; Yang et al., 2017; Duc et al., 2024). There is an urgent need for measures to reduce Internet addiction among college students.

Secondly, the present study found that Internet addiction was associated with lower life satisfaction, which was consistent with previous studies (Shahnaz and Karim, 2014; Şahin, 2016). Internet addicts were reported to experience profound academic problems, eventually resulting in poor grades, academic probation, and even expulsion from universities (Young, 1998), and the presence of these problems was unlikely to foster a sense of life satisfaction among students. In previous research, lower academic achievement has been shown to predict diminished life satisfaction (Chow, 2005), study-related depression and anxiety further erode students’ overall evaluations (Guney et al., 2010). These findings align with the present results and strengthen the argument that academic difficulties precipitated by excessive internet use undermine college students’ life satisfaction. In addition, the results found that Internet addiction did not affect life satisfaction directly, but partly through the mediating effect of perceived social support. The result coincides with the previous findings that Internet addiction was negatively correlated with perceived social support (Swickert et al., 2002; Naseri et al., 2015), and perceived social support was positively correlated with life satisfaction (Adams et al., 2016; Alorani and Alradaydeh, 2018). This finding can be interpreted to mean that the excessive use of Internet leads to social isolation and reduced relationships, which in turn leads to lower perceived social support. Further, low levels of social support are insufficient to help college students cope with the challenging college life, thus decreasing their life satisfaction.

Finally, we found that the indirect effect of Internet addiction on life satisfaction through social support was moderated by grit. Specifically, results showed that when grit level is low, the negative indirect effect of internet addiction on life satisfaction through social support is significant. However, when the grit level is higher, this negative mediating path is weakened or even no longer significant. In other words, the higher the grit level is, the more it can weaken the negative impact of internet addiction on life satisfaction through social support; on the contrary, when the grit level is low, the negative mediating effect is more prominent. These results further confirm the importance of developing grit, as it implies stronger self-control and goal-oriented persistence, which help students buffer the negative impact of Internet addiction on social relationships by persisting to maintain offline social connections (Liu et al., 2024). Notably, our analysis also revealed that the protective effect of grit was not uniformly significant across all levels: while grit effectively weakens the negative mediating path at low to moderate levels, its effect becomes non-significant when grit reaches moderate to high levels. This pattern may be attributed to two key factors. First, there is a potential “ceiling effect” of grit at moderate to high levels. According to Duckworth et al. (2007), grit (perseverance and passion for long-term goals) exerts a more prominent buffering effect when individuals face clear, manageable challenges. In our sample, college students with moderate to high grit already demonstrated strong resilience in coping with daily stressors (e.g., academic pressure, time management issues) related to internet addiction; when their grit level exceeded a certain threshold, the marginal gain from additional grit in improving life satisfaction became negligible, meaning further increases in grit did not lead to significant changes in the outcome variable. Second, the non-significant effect may reflect the complexity of the moderating mechanism. At moderate to high grit levels, other factors (e.g., social support quality) may have played a more dominant role in mitigating the negative impact of internet addiction on life satisfaction. This aligns with the complexity of the interactive nature of protective factors in well-being research. Therefore, this result indicated that we should particularly pay more attention to the low-grit students. In some cases, addictive behavior serves as a coping mechanism for young people having trouble negotiating their developmental challenges (Kandell, 1998); therefore, they appeared to rely more on the Internet for emotional compensation, leading to social isolation and reduced real-world support (Chou et al., 2005).

According to the results of the present study, the following suggestions may be put forward for college teachers and administrators to work on the negative influence of Internet addiction. First, educators should be aware of the growing tendency of Internet addiction among college students and understand the harmful effects of Internet addiction on life satisfaction. Second, considering the importance of support from parents, teachers, and friends in helping college students cope with academic and social challenges, support groups aiming at decreasing Internet dependence should be organized on the campuses and provide tailored intervention strategies and psychological counseling to those found addicted to develop a higher perception of social support. Third, both teachers and parents should be involved in developing programs to cultivate the quality of grit and interpersonal skills of Internet addicts to improve self-control ability and help them maintain healthy social relationships in their real lives.

Although the present findings provided important insights, several limitations should be considered. Firstly, the samples in this study were collected only in China, thus failing to illustrate a comprehensive picture of the prevalence of Internet addiction among college students. A larger scale of investigation, including college students from worldwide would improve the accuracy of this data. Secondly, this study was conducted in a cross-sectional way. It is recommended that future studies use a longitudinal design to examine and verify the cause-and-effect relationships between these variables. Lastly, the use of self-reported questionnaires may be accompanied by the problem of social desirability bias. For example, self-denial of problematic behaviors has been identified as one of the characteristics of Internet addicts. Thus, a more validated and effective diagnosis measure of Internet addiction should be developed or used in a similar research to avoid this bias and confirm the results.

## 6 Conclusion

To conclude, our study showed that Internet addiction influenced college students’ life satisfaction negatively by reducing their perceived social support, and grit played a moderated mediation role in that mechanism. This finding emphasizes the importance of taking actions to offset the adverse impact of Internet addiction on students’ life satisfaction. Since college students are the most vulnerable population to become addicted to Internet, and the addicted population has been growing rapidly, intervention programs and policies should be carried out urgently to control the situation. More importantly, this study helps us to understand that Internet addiction affects life satisfaction through decreasing their perceived social support; however, this mediating effect can be negated through fostering the quality of grit. Thus, efforts to boost perceived social support and grit for Internet addicts should focus on providing more support and coping strategies from not only colleges, but also from their families, and even our society.

## Data Availability

The raw data supporting the conclusions of this article will be made available by the authors, without undue reservation.
